# Conservation of core gene expression in vertebrate tissues

**DOI:** 10.1186/jbiol130

**Published:** 2009-04-16

**Authors:** Esther T Chan, Gerald T Quon, Gordon Chua, Tomas Babak, Miles Trochesset, Ralph A Zirngibl, Jane Aubin, Michael JH Ratcliffe, Andrew Wilde, Michael Brudno, Quaid D Morris, Timothy R Hughes

**Affiliations:** 1 Department of Molecular Genetics, University of Toronto, 160 College Street, Toronto, Ontario M5S 3E1, Canada; 2 Department of Computer Science, University of Toronto, 160 College Street, Toronto, Ontario M5S 3E1, Canada; 3 Banting and Best Department of Medical Research, University of Toronto, 160 College Street, Toronto, Ontario M5S 3E1, Canada; 4 Department of Immunology and Sunnybrook Research Institute, University of Toronto, 160 College Street, Toronto, Ontario M5S 3E1, Canada; 5 Terrence Donnelly Centre for Cellular and Biomolecular Research, University of Toronto, 160 College Street, Toronto, Ontario M5S 3E1, Canada; 6 Current address: Department of Biological Sciences, University of Calgary, 2500 University Drive NW, Calgary, Alberta, T2N 1N4 Canada; 7 Current address: Rosetta Inpharmatics, 401 Terry Avenue North, Seattle, WA 98109, USA

## Abstract

**Background:**

Vertebrates share the same general body plan and organs, possess related sets of genes, and rely on similar physiological mechanisms, yet show great diversity in morphology, habitat and behavior. Alteration of gene regulation is thought to be a major mechanism in phenotypic variation and evolution, but relatively little is known about the broad patterns of conservation in gene expression in non-mammalian vertebrates.

**Results:**

We measured expression of all known and predicted genes across twenty tissues in chicken, frog and pufferfish. By combining the results with human and mouse data and considering only ten common tissues, we have found evidence of conserved expression for more than a third of unique orthologous genes. We find that, on average, transcription factor gene expression is neither more nor less conserved than that of other genes. Strikingly, conservation of expression correlates poorly with the amount of conserved nonexonic sequence, even using a sequence alignment technique that accounts for non-collinearity in conserved elements. Many genes show conserved human/fish expression despite having almost no nonexonic conserved primary sequence.

**Conclusions:**

There are clearly strong evolutionary constraints on tissue-specific gene expression. A major challenge will be to understand the precise mechanisms by which many gene expression patterns remain similar despite extensive *cis*-regulatory restructuring.

## Background

Vertebrates all share a body plan, gene number and gene catalog [1-4] inherited from a common progenitor, but so far it has been unclear to what degree gene expression is conserved. King and Wilson [5] initially posited that phenotypic differences among primates are mainly due to adaptive changes in gene regulation, rather than to changes in protein-coding sequence or function, and this idea has accumulated supporting evidence in recent years [6-12]. Recent work has indicated that gene expression evolves in a fashion similar to other traits, where in the absence of selection, random mutations introduce variants within a population [11,13-19]. Changes negatively affecting fitness are probably eliminated by purifying selection: core cellular processes seem to be coexpressed from yeast to human [20], and conservation of the expression of individual genes in specific tissues has been observed across distantly related vertebrates [21-24], perhaps reflecting requirements for patterning and development as well as conserved functions of organs, tissues and cell types. Conversely, changes that benefit fitness (for example, under new ecological pressures) may become fixed: changes in gene expression are believed to underlie many differences in morphology, physiology and behavior and, indeed, subtle differences in gene regulation can result in spatial and temporal alterations in transcript levels, with phenotypic consequences at the cell, tissue and organismal levels [5,25]. The degree to which stabilizing selection constrains directional selection and neutral drift across the full vertebrate subphylum is, to our knowledge, unknown.

Comparative genomic analyses provide a perspective on the evolution of both *cis*- and *trans*-regulatory mechanisms, and they are often used as a starting point for the identification of regulatory mechanisms. One estimate, using collinear multiple-genome alignments, suggested that roughly a million sequence elements are conserved in vertebrates (particularly among mammals, which represent the majority of sequenced vertebrates) [26-29], with most being nonexonic [28], and a series of studies have demonstrated the *cis*-regulatory potential of the most highly conserved nonexonic elements (for example, [27,29,30]). Another study [31] found that only 29% of nonexonic mammalian conserved bases are evident in chicken, and that nearly all aligning sequence in fish overlaps exons, raising the possibility that gene regulatory mechanisms may be very different among vertebrate clades. Absence of conserved sequence does not imply lack of regulatory conservation, however, as many known *cis*-regulatory elements seem to undergo rapid turnover [32,33], and there are examples in which orthologous genes have similar expression patterns despite apparent lack of sequence conservation in regulatory regions [34]. As further evidence of pervasive regulatory restructuring in vertebrate evolution, an analysis [35] that accounted for shuffling (non-collinearity) of locally conserved sequences suggested that the number of conserved elements may be several fold higher than collinear alignments detect, particularly between distant vertebrate relatives, such as mammals and fish.

*Trans*-acting factors (transcription factors or TFs) also show examples of striking conservation, such as among the homeotic factors, and diversifying selection [36]. Studies comparing expression patterns between human and chimpanzee liver found that TF genes were enriched among the genes with greatest human-specific increase in expression levels [37,38], supporting arguments for alteration of *trans*-regulatory architecture as a driving evolutionary mechanism [39]. On the other hand, in the *Drosophila *developmental transition, expression of transcription factor genes is more evolutionarily stable than expression of their targets, on average [40]. The fact that enhancers will often function similarly in fish and mammals, even when the enhancer itself is not conserved, indicates that mechanisms underlying cell-specific and developmental expression are likely to be widely conserved across vertebrates [41,42].

Global trends in conservation of gene expression, conservation of *cis*-regulatory sequence and relationships between the two are not completely understood [13,39,41], partly because the *cis*-regulatory 'lexicon' (that is, how TF binding sites combine to form enhancers) remains mostly unknown, testing individual enhancers is tedious and expensive, and many vertebrates are not amenable to genetic experimentation. These issues are of both academic and practical consequence: in addition to our curiosity about the origin and distinctive characteristics of the human species, primary sequence conservation is widely used to identify regulatory mechanisms. We reasoned that expression profiling data from species spanning much greater phylogenetic distance than humans and mice, and thus having greater opportunity for both neutral drift and positive selection, would allow assessment of the degree of conservation of tissue gene expression among all vertebrates, and a comparison of the conservation of expression to the conservation of nonexonic primary sequence. Here, we describe a survey of gene expression in adult tissues and organs in the main vertebrate clades: mammals, avians/reptiles, amphibians and fish. Our analyses demonstrate that core tissue-specific gene expression patterns are conserved across all major vertebrate lineages, but that the correspondence between conservation of expression and amount of conserved nonexonic sequence is weak overall, at least at a level that is detectable by current alignment approaches.

## Results

### Tissue-specific gene expression is broadly correlated across vertebrates

To examine gene expression in a broad range of vertebrates, we collected a compendium of gene expression datasets, consisting of previously published datasets for human [43] and mouse [44], and newly generated datasets containing 20 tissues each from chicken (*Gallus gallus*), frog (*Xenopus tropicalis*) and pufferfish (*Tetraodon nigroviridis*). Details of the experiments are found in the Materials and methods; lists of tissues are found in Additional data file 1. Clustering analyses of each dataset separately (Additional data file 2) shows that prominent tissue-specific expression patterns are found in all vertebrates.

**Figure 1:**
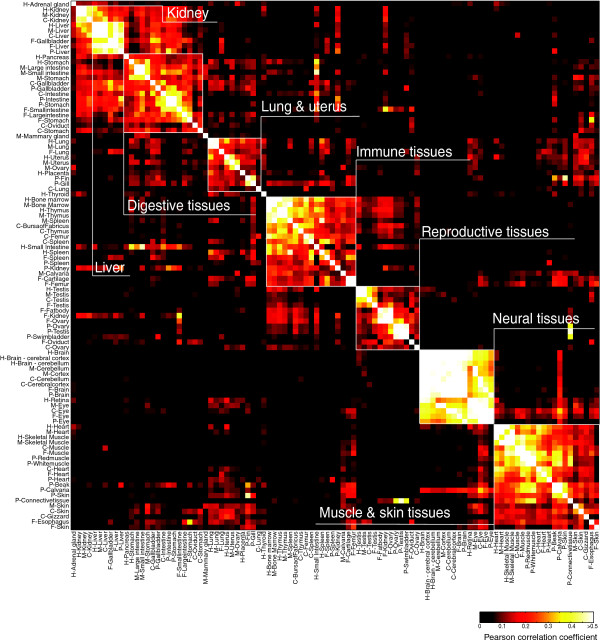
Comparison of tissue expression profiles among five diverse vertebrates. Clustered heat map of the all-versus-all Pearson correlation matrix between 20 tissues in each of human (H), mouse (M), chicken (C), frog (F) and pufferfish (P) over all 3,074 1-1-1-1-1 orthologs. Analogous and functionally related tissues are boxed in white, demonstrating the cross-species similarity of those tissues on the basis of their gene expression profiles.

To ask whether tissue-specific gene expression patterns are conserved among vertebrates, we focused on 1-1-1-1-1 orthologs (genes that are present in a single unambiguous copy in each of the five genomes), because genes that have undergone duplication events are subject to different constraints from singletons [45,46]. Among 4,898 1-1-1-1-1 orthologs found by Inparanoid [47], 3,074 were measured by microarrays in all ten common tissues of chicken, frog, pufferfish, and mammals (human and mouse combined expression – see Materials and methods). The expression profiles of these 3,074 genes in analogous and functionally related tissues in different species were more similar than they were to those of unrelated tissues from the same species (Figure 1), even for pufferfish, which diverged from the other vertebrates in our study roughly 450 million years ago (Mya), well before the divergence of frog (about 360 Mya) or chicken (about 310 Mya) [48]. Despite differences in cognition and behavior between humans and other species, overall gene expression in the brain is most similar across the species studied compared with expression in other tissues (median expression ratio Pearson correlation (*r*) = 0.63), consistent with a previous study comparing human and chimpanzee [49]. The relatively low divergence of gene expression in brain is hypothesized to be due to constraints imposed by the participation of neurons in more functional interactions than cells in other tissues [50]. In contrast, gene expression in the kidney was most dissimilar between species (median expression ratio Pearson *r *= 0.21), possibly reflecting evolution of kidney function (see Discussion). A dendrogram for the ten common tissues (with the same tissue measured in all five datasets; Additional data file 3) shows clear segregation of the data for heart/muscle, eye, central nervous system (CNS), spleen, liver and stomach/intestine. Only the testis and kidney datasets are split, each into two groups, with pufferfish and/or frog forming the outlying group. Additional data file 4 shows that, among these 3,074 genes, the Gene Ontology (GO) processes enriched in tissues are also generally conserved across the five species. We conclude that programs of tissue-specific expression are broadly conserved among vertebrates.

### Thousands of individual tissue-specific gene expression events are conserved across all vertebrate clades

**Figure 2:**
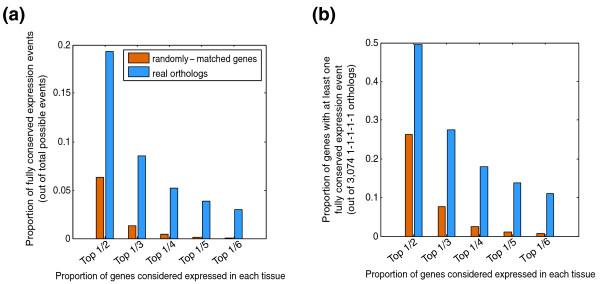
Conservation of gene expression using the binary measure. **(a) **Proportion of conservation events out of total possible conservation events at different thresholds using the binary model. **(b) **Proportion of genes with at least one conservation event among the ten common tissues out of all 3,074 measured genes using the binary model. See Results and Materials and methods for details.

We next sought to quantify the conservation of expression of individual genes. We used two conceptually simple measures intended to capture different aspects of conservation of expression. The first asks how often specific gene expression events (instances in which gene X is expressed in tissue Y) are conserved across all vertebrates. We refer to this as the 'binary measure' because, to simplify statistical analysis, we considered a fixed proportion of the normalized, ranked microarray intensities of genes in each tissue to be expressed ('1'), and analyzed the data using several such proportions (1/6, 1/5, 1/4, 1/3, 1/2; Additional data file 5 contains the binary matrices). We then asked how often a gene is expressed in all species in a given tissue (that is, a fully conserved expression 'event'). The proportion of conserved expression events at different thresholds ranges from 3% to 19.3% of all possible expression events, among the 3,074 1-1-1-1-1 orthologs (Figure 2a), and the proportion of genes with at least one conservation event ranges from 11% to 49.5% (Figure 2b), in all cases clearly exceeding permuted (negative control) datasets. On the basis of the spread between blue and orange bars in Figure 2, about 10% of the 30,740 possible gene expression events are conserved among all vertebrates, and at least 20% of all 1-1-1-1-1 orthologs participate in at least one such event. This measure probably underestimates the conservation of gene expression, because we surveyed only ten tissues and because we have not considered lack of expression across all species to represent an example of conserved expression.

**Figure 3:**
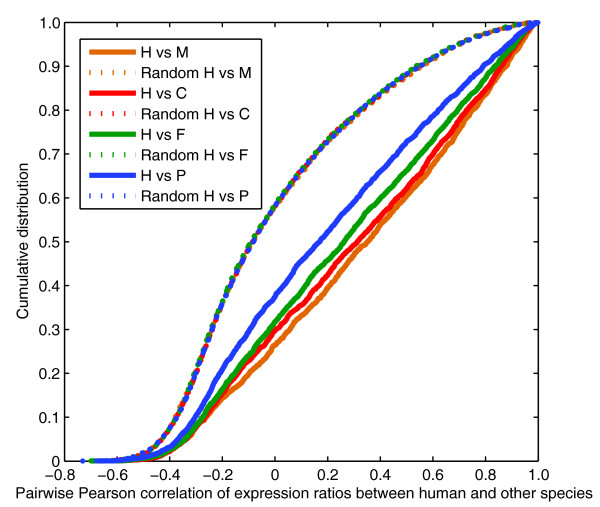
Cumulative distributions comparing the pairwise conservation of gene expression of each species versus human using the Pearson correlation measure. Data shown use median-subtracted asinh values (comparable to ratios). The dotted lines are negative controls derived using permuted data. C, chicken; F, frog; H, human; M, mouse; P, pufferfish.

The second measure we used was Pearson correlation across the ten common tissues. As with the binary measure, we found that gene expression across tissues between real 1-1-1-1-1 orthologs is more similar than randomly matched genes in pairwise comparisons between species (Figure 3 shows results for other species versus human; Additional data file 6 shows all pairwise comparisons, and also the median of pufferfish versus all other species, to provide a summary of overall conservation). The difference between the real and random (permuted) lines in Figure 3 and Additional data file 6 indicates that roughly 20% of all 1-1-1-1-1 orthologs display conserved expression – a proportion comparable to that obtained using the binary measure. In fact, at *r *= 0.4, the apparent false discovery rate is similar to that obtained with the 1/3 cutoff using the binary measure (27.4% versus 34.5%), as is the number of genes classified as having conserved expression (843 versus 1,062). The overlap between these two sets of genes is higher than expected at random (417 versus 291 at random); however, it is far from absolute, indicating that the definition of conserved expression influences conclusions regarding conservation of expression.

**Figure 4:**
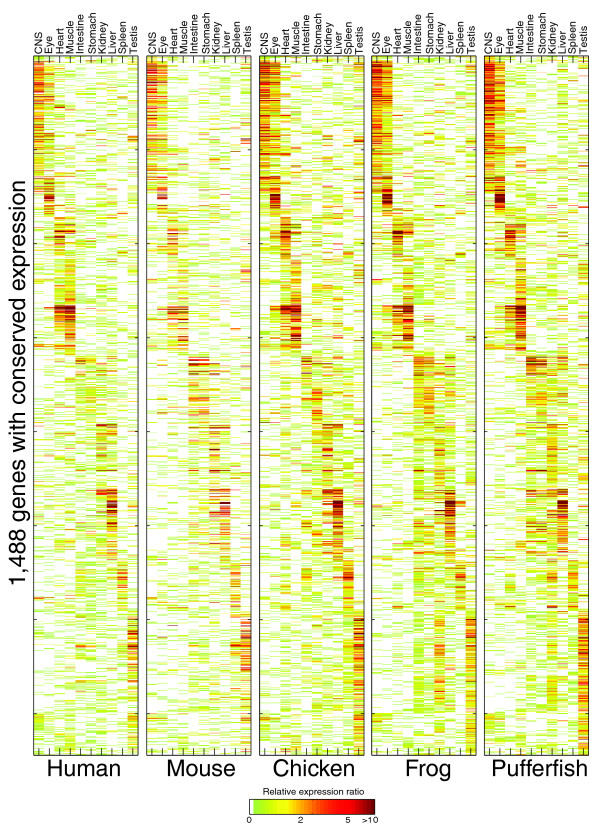
A core conserved vertebrate tissue transcriptome. Expression ratios of the measured and predicted expression patterns of 1,488 1-1-1-1-1 orthologs as described in the text and Materials and methods are shown. Two-dimensional hierarchical agglomerative clustering using a distance metric of 1 – Pearson correlation followed by clustering and diagonalization [44] was applied to the expression ratios of each ortholog in each tissue over all five datasets.

Regardless of the method of comparison the same essential conclusion is reached: a major component of tissue gene expression has apparently remained intact since the common ancestor of all vertebrates. A large fraction of genes is encompassed; between the two measures (the binary measure and the Pearson measure), 48.4% of all 1-1-1-1-1 orthologs (1,488/3,074) scored as having conserved expression at about 30% apparent false discovery rate. Thus, in just the ten common tissues we analyzed, gene expression is at least partially conserved for at least a third of all unique orthologs (48.4% × 0.7 = 33.9%) by at least one of our two definitions of conservation. The expression of these 1,488 genes in modern-day lineages is shown in Figure 4. Most of these genes have tissue-specific patterns of expression, indicating that the genes we are identifying are not simply ubiquitously expressed housekeeping genes.

**Figure 5:**
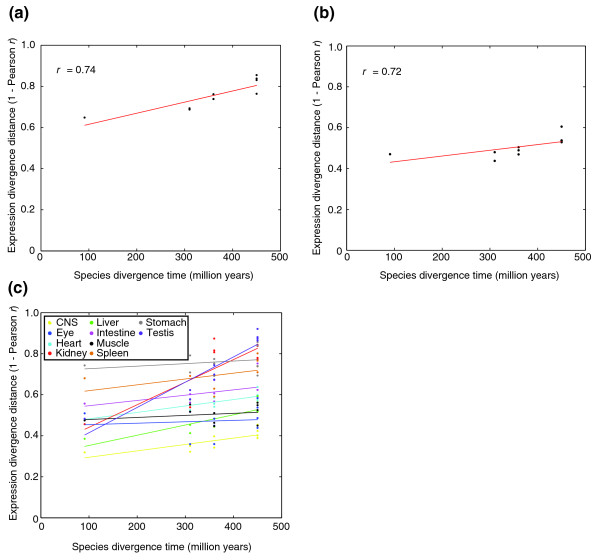
Comparison of gene expression conservation to evolutionary distance. The scatter plots show expression distance as 1 – Pearson correlation, using median-subtracted asinh values (comparable to ratios). **(a) **Median pairwise correlation over all genes; each point represents a pair of species. **(b) **Median pairwise correlation over all tissues; each point represents a pair of species. **(c) **Individual pairwise correlations over tissues, as indicated with colors; each point represents a single tissue in a single pair of species. Estimated species divergence times were obtained from [48].

Although the focus of our study was to identify conserved gene expression patterns, our data are consistent with previous findings that divergence of gene expression scales with evolutionary time [17,18] when averaged over all genes (Figure 5a) or all tissues (Figure 5b; the same trend is apparent in Figure 4 and Additional data file 3). Individual tissue expression profiles show different evolutionary trajectories, however (Figure 5c), presumably reflecting diversity in constraints on tissue function.

### Conservation of expression does not correlate with proportion or amount of conserved nonexonic sequence

We next asked what gene properties correlate with conservation of expression among the 3,074 measured unique orthologs. We considered the following gene properties: those that are contained in our data, that is, median expression level and Shannon entropy as a measure of tissue specificity and preferential expression in individual tissues; GO annotations; and sequence properties, that is, length of gene, size of encoded protein, presence of a DNA-binding domain (for known and predicted TFs), sequence conservation of encoded protein (pairwise BLASTP bit score) and amount of conserved nonexonic sequence (measured in several ways) (Additional data files 7 and 8; see Materials and methods for details).

Several observations emerged from this analysis. First, the genes with the highest expression similarity between species are most often genes expressed in a highly tissue-specific manner in tissues with specialized functions. Although the Pearson correlation is heavily influenced by extreme values, thus giving higher weight to tissue-specific pairs, most of these high scoring genes were also classified as conserved by our binary measure. Among the 50 genes with highest median pairwise Pearson correlation of expression are structural components of the eye lens, liver-synthesized proteins involved in the complement system and blood coagulation, and neurotransmitter receptors and transporters. This observation is supported by the GO categories enriched among genes with high expression similarity, such as synaptic transmission (GO:0007268), visual perception (GO:0007601), wound healing (GO:0042060) and muscle development (GO:0007517) (Wilcoxon-Mann-Whitney test (WMW) *p*-values 1.55 × 10^-4^, 2.36 × 10^-3^, 2.24 × 10^-3 ^and 4.98 × 10^-5^, respectively; Additional data file 8). In contrast, we did not find any evidence that the expression of TFs (228 of the 3,074 measured orthologs) is more or less conserved than that of non-TFs, in contrast to previous reports of both higher [38] and lower [40] rates of evolution of TF expression. A slightly lower proportion of TFs did seem to show conservation events relative to non-TFs using the binary measure, but this difference is due to the fact that TFs are expressed in fewer tissues: the difference is not seen when comparing TFs and non-TFs with similar overall expression levels (data not shown).

**Figure 6:**
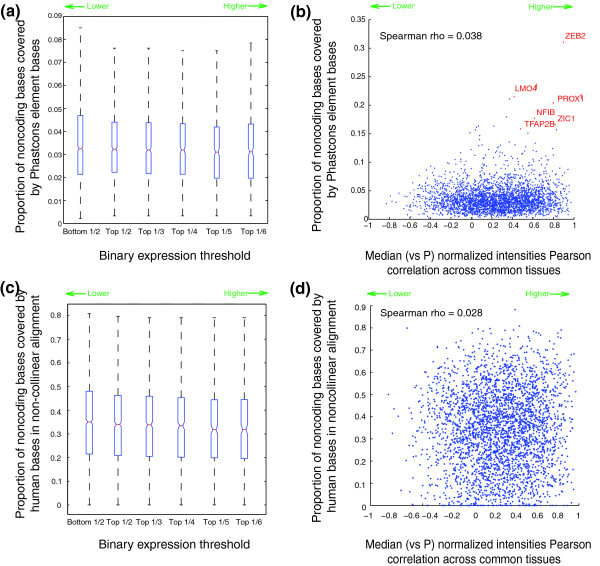
Relationship between expression similarity between orthologous genes and amount of conserved nonexonic sequence. Proportion of conserved nonexonic sequence defined as Phastcons elements **(a, b) **and human bases in non-collinear alignment **(c, d) **compared against the conservation of gene expression by the binary measure (a, c) and Pearson measure (normalized intensities) versus pufferfish (P) (b, d) (see text and Materials and methods for details). Selected TFs are indicated in (b) (see text). Probable TFs as determined by their Ensembl gene descriptions, but that were not identified by our domain analyses, are indicated by †. Spearman rho refers to the Spearman correlation coefficient.

It is widely believed that conserved nonexonic sequence often serves a *cis*-regulatory function, and it follows that a larger amount of conserved nonexonic sequence might correlate with a higher probability of conserved expression. However, we found that the correspondence was very weak: for example, for the binary model, we obtained Spearman correlations of -0.086 and 0.0029 with the number of nonexonic bases in Phastcons conserved regions [28] and in ultraconserved elements (UCEs) [26], respectively; for the Pearson model, these correlations were 0.054 and 0.0075, respectively. Similar results were obtained when proportion of bases replaced number of bases (Figure 6a,b). The handful of outlying points in the upper right of Figure 6b includes several TFs, a subset of which are known to have an exceptional degree of nonexonic sequence conservation [26].

We reasoned that pervasive shuffling might obscure most of the *cis*-regulatory elements, particularly in pufferfish. In order to address this possibility, we developed a technique similar to that of Sanges *et al. *[35] to detect shuffled conserved sequence elements (SCEs), which may be non-collinear, across the five species (see Materials and methods for details). Among the total 4,898 1-1-1-1-1 orthologs, we identified 491,028, 457,074, 79,001, 54,134 and 11,731 SCEs in human, mouse, chicken, frog and pufferfish with median lengths of 164, 80, 68, 68 and 65 nucleotides, respectively. These SCEs showed good overlap with those in [35] (75.5% of the sequences in [35] within regions we aligned were identified as SCEs in our analysis) and they were calibrated to minimize false positives (see Materials and methods). However, we still did not observe a strong relationship between the degree of conservation and the proportion or number of aligned bases in each species (median Spearman correlation: -0.062 and 0.042 for binary and Pearson models, respectively, versus proportion of aligned nonexonic bases in each species; Figure 6c,d; similar correlations are obtained with number of aligned non-exonic bases).

**Figure 7:**
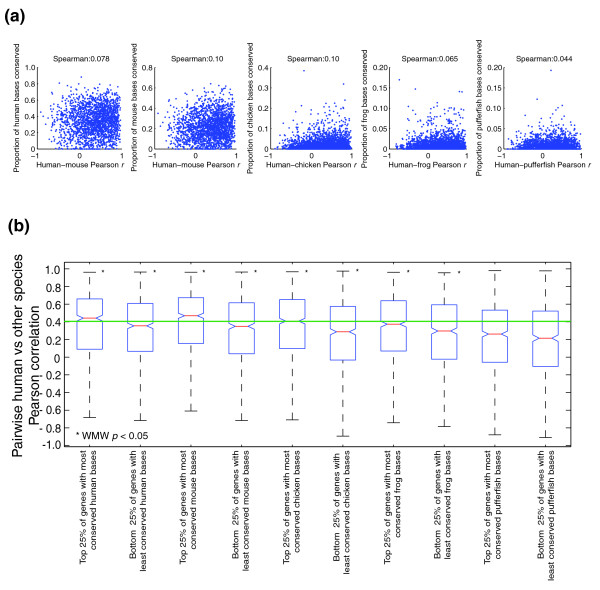
Low correlation between conservation of gene expression and amount of conserved nonexonic sequence is largely independent of evolutionary distance. **(a) **Scatter plots show the proportion of bases conserved in SCE alignments versus Pearson correlation (ratios) for individual genes. **(b) **Box plots show the distribution of Pearson correlations for genes in the top and bottom quartiles of number of conserved bases. Asterisks indicate significant differences between the top and bottom quartiles.

**Figure 8:**
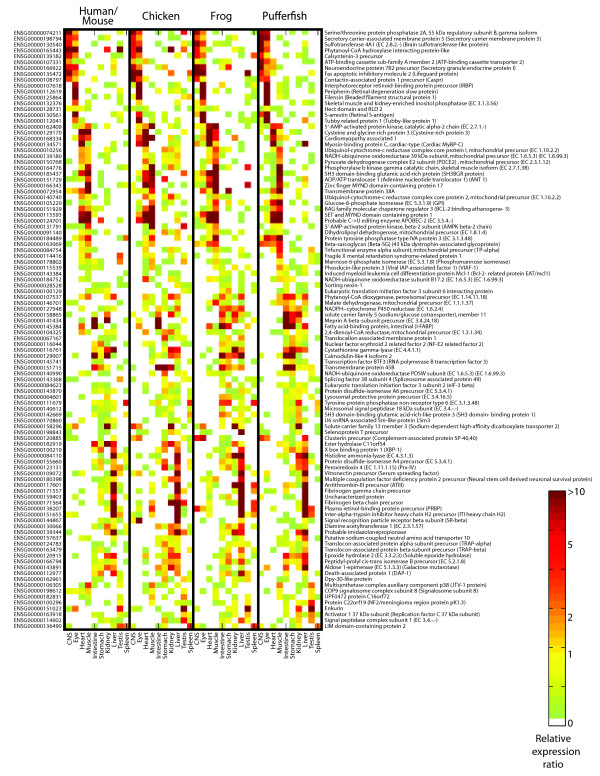
Expression of 102 genes with highly conserved expression across all vertebrate lineages, but no detectable nonexonic sequence conservation between pufferfish and frog, chicken, mouse, or human. Mouse and human expression profiles are merged to represent mammals. Gene identifiers and descriptions for human were downloaded from Ensembl.

We also examined the correlations between nonexonic sequence conservation and expression correlation at varying evolutionary distances from human. Although correlations remain weak (Figure 7a), we did find that genes in the highest quartile of sequence conservation had a significantly higher distribution of expression correlation than those in the lowest quartile, for all pairwise comparisons except human versus pufferfish (Figure 7b). However, in all comparisons, there are many genes with little sequence conservation and high expression correlation, and vice versa. In fact, among the 173 genes with the most highly conserved expression in our study by both measures we applied (those in the top 1/6 by the binary measure and with median Pearson *r *≥ 0.5), most (102) have no nonexonic conserved sequence in fish, on the basis of our SCEs. The expression of these 102 genes in the ten common tissues in the representatives of all modern lineages is shown in Figure 8.

Because TF binding sites are degenerate, it is conceivable that these genes have a high number of conserved TF binding sites, despite their lack of primary sequence conservation. To examine this possibility we used Enhancer Element Locator (EEL) [51] to align TF binding sites defined by 138 motif models downloaded from the JASPAR database [52]. Over all 4,804 aligned human/pufferfish ortholog pairs, the number of genes that scored highly using EEL was only slightly higher with real ortholog pairs than with randomly assigned orthologs with similar amounts of nonexonic associated sequence in both genomes (*p *= 0.24, Kolmogorov-Smirnov test; see Materials and methods and Additional data file 9) and there is almost no correlation between EEL score and conservation of expression (EEL score against median versus pufferfish normalized intensity Pearson *r *= 0.022). We conclude that the regulatory architecture of the vast majority of genes has diverged beyond recognition by any current approaches, despite the apparently very similar regulatory output in many cases, and the likelihood that at least some orthologous TFs are functioning in the same tissues.

## Discussion

Our data provide a resource of large-scale gene expression data in tissues of three non-mammalian vertebrates and demonstrate that there is conservation of core vertebrate tissue gene expression. Our analysis almost certainly underestimates the proportion of genes with conserved expression patterns, because our analysis focused on only ten large adult tissues in mature animals in captivity. Nonetheless, our results already provide an index of several thousand highly conserved tissue gene expression events and a picture of core gene expression in major tissues and organs of the common progenitor of all vertebrates, which most likely resembled the expression patterns shown in Figure 4.

In our analysis, some biological processes emerged as more highly conserved than others. Genes involved in the more conserved processes on the whole tend to be preferentially expressed in tissues with a limited number of cell types (brain, eye, liver, heart and muscle) that carry out specialized functions particular to those tissues. This finding is consistent with the notion that mechanisms underlying important biological processes should be conserved across taxa [53]. It is likely that the conservation of gene expression in these tissues extends beyond the base of vertebrates; coexpression of neuronal genes, for example, is observed as far as nematodes [54]. Genes expressed in tissues subject to greater environmental influence (such as intestine, stomach and spleen) may be more likely to take on new roles and diverge in expression as means of adaptation. We find gene expression similarity in the testis across vertebrates to be relatively low compared with other tissues, in support of earlier observations of accelerated evolution of testis gene expression in *Drosophila *[55] and primates [49], and consistent with mating competition and speciation. We also note that relatively low conservation of gene expression in the kidney is consistent with its divergent function; in teleosts, the kidney is a major lymphoid organ [56].

Our finding that the correlation between the amount of conserved nonexonic sequence and conservation of gene expression is low underscores the apparent malleability of the *cis*-regulatory 'lexicon' [32,35,57]. It is easy to rationalize cases in which there is conserved sequence but little or no conservation of expression in our study, because it is possible that the majority of conserved sequence identified is either not *cis*-regulatory, is functioning as *cis*-regulatory in a context that we have not measured and/or regulates neighboring genes. What is most striking is that many genes with the highest conserved tissue-specific expression have almost no nonexonic conserved sequence outside mammals. Divergence in *trans*-regulatory architecture does not provide a satisfying explanation for this observation: although there are examples in which the binding specificity of TFs evolves [58,59], as a general rule the individual monomeric TF sequence specificities seem to be unchanged over large evolutionary distances [60], and DNA-contacting residues are often the most conserved [61]. The fact that conservation of TF expression is comparable to that of other genes with similar levels of tissue specificity also supports the notion that many ancestral vertebrate regulatory mechanisms are still in use. Moreover, enhancers can be functional across large evolutionary distances (for example, human reporters in zebrafish), even when the enhancers are not conserved, or are below the threshold of detection by current alignment techniques [42].

Understanding the mechanisms underlying conservation of expression patterns is a major challenge in our understanding of evolution, and of genome function and gene regulation: even within a single genome, it is difficult to find *cis*-regulatory modules shared by coexpressed genes, indicating that there are many ways to achieve the same expression output. We propose that our catalog of conserved (and non-conserved) expression will be useful to test ideas regarding enhancer definition. In particular, we predict that the small size of pufferfish genes and knowledge of the expression of TFs in each tissue may facilitate searches for enhancers on the basis of density and arrangement of TF binding sites, rather than primary sequence alignment [34,62]. These and other techniques require experimental data for training and testing, and we now provide such data for tens of thousands of genes across the vertebrate lineage, including several thousand unique orthologs.

## Materials and methods

### Data availability

Data tables including microarray data, probe sequences, the 1-1-1-1-1 ortholog list, Phastcons information, UCE information, GO annotations and novel non-collinear alignments are found in the Additional data files and also on our project website [63].

### Tissue sources

Five chickens (three young females, one in-lay adult female and one rooster) were obtained from Sunnybrook Health Sciences Centre, Toronto, Canada. The bursa of Fabricius and thymus were dissected from the young females. The oviduct and ovaries were dissected from the in-lay adult female and the testis dissected from the rooster. Approximately 75 adult female and five adult male frogs were obtained from Nasco (Atkinson, USA), housed in 20 L aquariums at 20°C and fed a diet of Aquamax Grower 600 trout food (Purina Mills, Gray Summit, USA). Exactly 100 green spotted pufferfish, of unknown age and sex, described as caught wild in Malaysia, were purchased from Aquarium Services Warehouse Outlets (Thornhill, Canada) and housed in a single 120 gallon (453 L) aquarium tank at room temperature. Their markings, size (about 6–7 cm) and behavior matched descriptions of *Tetraodon nigroviridis*. The fish were fed frozen brine shrimp twice a day.

### Tissue collection and mRNA isolation

Animal handling and euthanization procedures were performed according to protocols approved by the University of Toronto. Tissue processing and mRNA extraction were as described previously [44]. Tissues collected are listed in Additional file 1. All fish and frogs were anaesthetized by adding 35 ml of a 1:10 clove oil (Rougier Pharma, Mirabel, Canada):ethanol mixture to 1.5 L tank water containing a single animal. Following cessation of movement (15–20 s), they were euthanized by decapitation. Chickens were euthanized by barbiturate injection followed by decapitation.

### Microarray design, processing and data collection

Oligonucleotide microarrays (60-mer) were custom-designed by us and manufactured by Agilent Technologies to survey the expression of a set of known and predicted mRNAs compiled from Ensembl [64] version 37 and JGI (for *X. tropicalis*, v3.0) corresponding to 24,877, 25,594 and 25,937 known and predicted loci in *Tetraodon*, chicken and *X. tropicalis*, respectively, using 41,533, 41,534 and 41,523 probes. cDNA synthesis, labeling reactions and microarray processing procedures were performed as described previously [44]. Each tissue was assayed in duplicate, in fluor-reversed pairs with biological replicates and averaged (average Pearson correlation between replicate arrays = 0.89). Using a previously described procedure to define expression [44], 72%, 77% and 89% of genes on the arrays were detected in at least one of the tissues profiled in chicken, frog and pufferfish, respectively, with 40%, 33% and 52% of genes detected in each tissue, on average (Additional data file 10), comparable to previously reported figures of 52% and 59% in human and mouse [44,65]. Our percentages may be lower because many predicted genes were included on the arrays. Microarray-based expression data for 55 mouse tissues [44] and 60 human tissues [43] were downloaded and mapped to Ensembl v37 identifiers by aligning probe sequences to Ensembl transcript sequences using BLAT [66]. Expression similarity was calculated using the relative expression levels or 'ratios' (median-subtracted, variance stabilization-normalized asinh intensities) as well as using the absolute values (normalized intensities) in each tissue, as indicated in the Results and figures. All new microarray data have been uploaded to the Gene Expression Omnibus (GEO) database, accession numbers [GEO:GSE12974, GEO:GSE12975, GEO:GSE12976], and are also found on our project website [63].

### Determination of gene orthology relationships

Inparanoid [67] was used to analyze each possible pairwise all-versus-all protein BLAST [68] comparison between all known proteins for each of the five vertebrate species in version 37 of the Ensembl database to delineate pairwise gene orthology relationships. These relationships were then assembled into unique 1-1-1-1-1 ortholog groups across the five species using custom Perl scripts in an approach analogous to that of Alexeyenko *et al. *[69].

### Gene Ontology analysis

GO annotations were downloaded from Ensembl BioMart [70] for each species. Annotations for chicken, frog and pufferfish (*Tetraodon*) were further supplemented by mapping the corresponding mouse annotations by any type of orthology as defined by Ensembl. Annotations were up-propagated and terms with few or too many annotated genes were removed as described previously [44]. All GO WMW analyses performed across the set of unique ortho-logs were done with human annotations.

### Definition of expression conservation events in each gene and tissue using the binary measure

We split each set of orthologs in each tissue in each species on the basis of their measured normalized expression intensities according to the following thresholds: top 1/2, top 1/3, top 1/4, top 1/5 and top 1/6. Because the human and mouse datasets were designed independently from those of the other three species, there were orthologous genes with missing measurements. In order to facilitate comparison between as many unique orthologs between the five species as possible, we applied variance stabilizing normalization [71] to the human and mouse orthologs in order to make them comparable, under the assumption that gene expression is mostly conserved between the mammals relative to the rest of the vertebrate phylogeny. We combined the human and mouse ortholog data by averaging to obtain a set of 3,074 unique orthologs with measured microarray data across ten common tissues.

### Calculating Shannon entropy as a measure of tissue specificity

Shannon entropy, which measures the degree of overall tissue specificity of a gene, was calculated as described by Schug *et al. *[72]. Briefly, the relative expression of a gene *g *in a tissue *t *relative to its expression given in *N *tissues is defined as *p*_*t*|*g *_= *w*_*g*, *t*_/Σ_1 ≤ *t *≤ *N *_*w*_*g*, *t*_, where *w*_*g*, *t *_is the expression level of the gene *g *in tissue *t*. The Shannon entropy of a gene's expression distribution is then calculated as *H*_*g *_= Σ_1 ≤ *t *≤ *N *_-*p*_*t*|*g*_log_2_*p*_*t*|*g*_. This value is expressed in bits and ranges from zero to log_2_(10) genes expressed in a single tissue and uniformly expressed in all the common tissues examined, respectively.

### Definition of overlap between nonexonic regions associated with human 1-1-1-1-1 orthologs and Phastcons elements and UCEs

Locations of Phastcons elements [28] and UCEs [26] were downloaded from the hg17 version of the UCSC genome browser [73] and from the supplementary website [74] of Bejerano *et al. *[26]. The number of Phastcons elements and UCEs (and the number of bases) that overlapped all nonexonic sequences, including intergenic sequences, up to 50 kb upstream and downstream of each human 1-1-1-1-1 ortholog was tabulated using custom Perl scripts. The proportion of Phastcons and UCE bases covered in non-coding regions was calculated as the number of bases out of 50 kb of flanking bases upstream and downstream of each gene with coding regions masked out.

### Definition and calculation of other gene features and statistical comparisons to expression conservation

A measure of protein sequence conservation between two species was derived by performing pairwise BLASTP [68] between the protein-coding sequences (downloaded from Ensembl EnsMart [70]) of an orthologous gene pair and retaining the bit score. The median bit score was taken as a measure of protein sequence conservation over all species. Average and maximum expression level and the expression rank within tissues were calculated in the expected manner for each gene across the ten common tissues. The number of bases in an aligned conserved element (aligned as described below) was obtained by summing the number of bases in each species within a five-way gapped alignment between all species. The total number of aligned bases in an aligned conserved element is the sum of counts in each species. TF genes within the set of unique orthologs were defined by the presence of a DNA-binding domain in the mouse protein sequence in the Pfam database [75]. Our list of TF genes is given in Additional data file 11. Conservation of expression was measured by either our binary or Pearson measures, both of which yield a real value (an integer between zero and six in the case of the binary method). With the exception of GO annotations and TF identities, all measures for each gene are compiled in Additional data file 7; TFs are listed separately in Additional data file 11. Comparisons of properties with real values were made by Spearman *p*-value, and these are also given in Additional data file 7 and shown graphically in Additional data file 12. Comparisons of categorical properties were made by WMW *p*-value and are given in Additional data file 8.

### Multiple sequence alignment algorithm

All repeat-masked intronic, 3' untranslated region and intergenic non-coding sequence upstream and downstream of protein-coding sequences in orthologous groups that we have identified using the Inparanoid algorithm [47] were downloaded from Ensembl v37 [64]. Using LAGAN [76], we built a multiple global alignment across all of the genomic sequences within each set of unique orthologs to identify conserved non-coding elements in all species. We used up to 50 kb of upstream and downstream sequence, up to the point that the transcript of another annotated gene was encountered.

We initially applied a conservation cutoff of 55% in a 50 nucleotide window to search for conserved regions between the human and the orthologous genomes. After removing conserved elements that were annotated as exonic, we extracted each sequence element in the alignment that was conserved only in a subset of the genomes and built the most parsimonious ancestral reconstruction of each of the sequences using Fitch's algorithm, treating the gap character as a fifth symbol. This was then used to search against the other orthologous genome(s) using the CHAOS aligner [77] with very sensitive parameters. The Smith-Waterman threshold was varied between 40 and 60 (60 is the default conservation setting for the BLASTZ [78] alignment program; this setting is both relatively sensitive and very specific, and thus almost all hits above this threshold will be real). This homology filtering step was used to identify non-collinear conserved sequences that may have changed position and orientation relative to exons over evolutionary time. In contrast to the method used by Sanges *et al. *[35], we tried using both the mouse and the chicken sequence as the homology filter. By using the chicken as the base organism we were able to significantly lower the false positive rate (only one decoy (i.e. permuted gene identity) sequence with a score above 45), while our true positive rate was unchanged (all of the human/frog and human/pufferfish conserved regions recovered with mouse as the base were also recovered with pufferfish (*Tetraodon*) as the base).

Through the procedure described above, our approach takes advantage of the conservation of order of the conserved elements when no rearrangements have taken place, and the flexibility of aligning less conserved regions that have been shuffled around in the genome. By running Fitch's algorithm on the aligned sequences (unlike the Sanges *et al. *[35] approach of simply using the mouse sequence as input to CHAOS) we increased the sensitivity of our alignment technique. In particular, it was easier to align *Tetraodon *sequence to the common ancestor of human and chicken genomes than to either genome individually.

### Enhancer Element Locator

We scanned the nonexonic sequence associated (as defined for the multiple sequence alignment algorithm) with 4,804 human/fish ortholog pairs for conserved TF binding sites by applying EEL [51] using the default parameters to perform a local pairwise TF binding site alignment. We did not attempt to align the 94 (of 4,898) ortholog pairs for which one of the orthologs appears in the intron of another gene. The motif models input into EEL were the 138 models returned by JASPAR webserver [52] on 27 February 2009. The score of only the best alignment from each orthologous gene group was captured and was used to construct a distribution of 4,804 gene group alignment scores. We also constructed a shuffled (negative control) distribution of EEL scores by attempting to align the TF binding sites between the human non-coding sequences in each of the ortholog pairs and those of six non-orthologous *Tetraodon *genes. These genes were selected among the other 4,803 non-orthologous paired *Tetraodon *genes to be the six with the most similar amount of non-coding sequence as the proper ortholog, under the constraint that three of the genes had to have less non-coding sequence and three had to have more non-coding sequence. As evidence that the EEL analysis is detecting some very limited degree of conservation, we note that the distribution of EEL scores is slightly higher in real than in randomly assigned orthologs starting around an EEL score of 175; in particular, we estimate that 1.3% (63 of 4,084) of all EEL scores are non-random by subtracting the proportion of random scores above 175 (39.8%) from the proportion of real scores above 175 (41.1%).

## Additional data files

The following additional data files are available. Additional data file 1: Tissues analyzed in this study. The tissues at the top, highlighted in color, are those considered to be among the ten common tissue types. Those with identical coloring were combined (by averaging normalized intensities) for the analysis of conservation of gene expression among the ten common tissues. Additional data file 2: Microarray gene expression data obtained in this study. Clustergrams show the microarray datasets in chicken, frog and pufferfish, displayed as relative expression ratio (see Materials and methods) of each gene within each of the 20 tissues profiled. Rows and columns were ordered independently for each dataset, and high-level branches broken and rearranged to obtain a diagonal appearance as described in [44]. Additional data file 3: Dendrogram of correlations among ten common tissues, using 1 – Pearson correlation and average linkage over 3,074 genes. Additional data file 4: Gene Ontology categories that tend to be expressed highly in each of the ten common tissues. Selected GO biological process categories enriched amongst genes highly expressed within each of the ten common tissues in each species are shown. The tissue and GO category order were manually arranged in the heat map. (A full matrix of WMW scores is given in Additional data file 13.) Additional data file 5: Binary matrix of genes classified as having fully conserved expression events, based on ranked microarray spot intensity, at five different thresholds (1/6, 1/5, 1/4, 1/3, 1/2). Additional data file 6: Cumulative distributions summarizing pairwise comparisons of conservation of gene expression using the Pearson correlation measure. The cumulative distributions show the proportion of all 3,074 genes with Pearson *r *(normalized intensities) below the value shown on the horizontal axis, for real orthologs (green) and randomly matched genes (blue). Additional data file 7: Feature matrix used to compare conservation of expression measures to other attributes of individual genes, with Spearman correlations and *p*-values. Additional data file 8: WMW *p*-values for categorical gene attributes, with ranks determined by relative conservation of gene expression by median Pearson correlation for each species against *Tetraodon*. Additional data file 9: Cumulative distribution of EEL scores for real and permuted orthology between human and pufferfish. Additional data file 10: Breakdown of the proportion of all genes in each species that are expressed within each tissue. Additional data file 11: List of genes classified as TFs on the basis of containing a known DNA binding domain. Additional data file 12: Clustergrams showing Spearman correlations and *p*-values for comparisons of gene expression conservation versus other gene properties. Additional data file 13: WMW enrichment *p*-values of genes associated with GO biological process annotations expressed within each tissue of each species (full matrix used to create Additional data file 4).

## Supplementary Material

Additional data file 1 The tissues at the top, highlighted in color, are those considered to be among the ten common tissue types. Those with identical coloring were combined (by averaging normalized intensities) for the analysis of conservation of gene expression among the ten common tissues. Click here for file

Additional data file 2 Clustergrams show the microarray datasets in chicken, frog and pufferfish, displayed as relative expression ratio (see Materials and methods) of each gene within each of the 20 tissues profiled. Rows and columns were ordered independently for each dataset, and high-level branches broken and rearranged to obtain a diagonal appearance as described in [44]. Click here for file

Additional data file 3 Dendrogram of correlations among ten common tissues, using 1 – Pearson correlation and average linkage over 3,074 genes. Click here for file

Additional data file 4 Selected GO biological process categories enriched amongst genes highly expressed within each of the ten common tissues in each species are shown. The tissue and GO category order were manually arranged in the heat map. (A full matrix of WMW scores is given in Additional data file 13.) Click here for file

Additional data file 5 Binary matrix of genes classified as having fully conserved expression events, based on ranked microarray spot intensity, at five different thresholds (1/6, 1/5, 1/4, 1/3, 1/2). Click here for file

Additional data file 6 The cumulative distributions show the proportion of all 3,074 genes with Pearson *r *(normalized intensities) below the value shown on the horizontal axis, for real orthologs (green) and randomly matched genes (blue). Click here for file

Additional data file 7 Feature matrix used to compare conservation of expression measures to other attributes of individual genes, with Spearman correlations and *p*-values. Click here for file

Additional data file 8 WMW *p*-values for categorical gene attributes, with ranks determined by relative conservation of gene expression by median Pearson correlation for each species against *Tetraodon*. Click here for file

Additional data file 9 Cumulative distribution of EEL scores for real and permuted orthology between human and pufferfish. Click here for file

Additional data file 10 Breakdown of the proportion of all genes in each species that are expressed within each tissue. Click here for file

Additional data file 11 List of genes classified as TFs on the basis of containing a known DNA binding domain. Click here for file

Additional data file 12 Clustergrams showing Spearman correlations and *p*-values for comparisons of gene expression conservation versus other gene properties. Click here for file

Additional data file 13 SWMW enrichment *p*-values of genes associated with GO biological process annotations expressed within each tissue of each species (full matrix used to create Additional data file 4). Click here for file

## References

[B1] International Chicken Genome Sequencing Consortium (2004). Sequence and comparative analysis of the chicken genome provide unique perspectives on vertebrate evolution. Nature.

[B2] International Human Genome Sequencing Consortium (2004). Finishing the euchromatic sequence of the human genome. Nature.

[B3] Jaillon O, Aury JM, Brunet F, Petit JL, Stange-Thomann N, Mauceli E, Bouneau L, Fischer C, Ozouf-Costaz C, Bernot A, Nicaud S, Jaffe D, Fisher S, Lutfalla G, Dossat C, Segurens B, Dasilva C, Salanoubat M, Levy M, Boudet N, Castellano S, Anthouard V, Jubin C, Castelli V, Katinka M, Vacherie B, Biémont C, Skalli Z, Cattolico L, Poulain J (2004). Genome duplication in the teleost fish Tetraodon nigroviridis reveals the early vertebrate protokaryotype. Nature.

[B4] Waterston RH, Lindblad-Toh K, Birney E, Rogers J, Abril JF, Agarwal P, Agarwala R, Ainscough R, Alexandersson M, An P, Antonarakis SE, Attwood J, Baertsch R, Bailey J, Barlow K, Beck S, Berry E, Birren B, Bloom T, Bork P, Botcherby M, Bray N, Brent MR, Brown DG, Brown SD, Bult C, Burton J, Butler J, Campbell RD, Mouse Genome Sequencing Consortium (2002). Initial sequencing and comparative analysis of the mouse genome. Nature.

[B5] King MC, Wilson AC (1975). Evolution at two levels in humans and chimpanzees. Science.

[B6] Cooper TF, Rozen DE, Lenski RE (2003). Parallel changes in gene expression after 20,000 generations of evolution in *Escherichia coli*. Proc Natl Acad Sci USA.

[B7] Ferea TL, Botstein D, Brown PO, Rosenzweig RF (1999). Systematic changes in gene expression patterns following adaptive evolution in yeast. Proc Natl Acad Sci USA.

[B8] Gompel N, Prud'homme B, Wittkopp PJ, Kassner VA, Carroll SB (2005). Chance caught on the wing: *cis*-regulatory evolution and the origin of pigment patterns in *Drosophila*. Nature.

[B9] Miller CT, Beleza S, Pollen AA, Schluter D, Kittles RA, Shriver MD, Kingsley DM (2007). *cis*-Regulatory changes in Kit ligand expression and parallel evolution of pigmentation in sticklebacks and humans. Cell.

[B10] Prud'homme B, Gompel N, Rokas A, Kassner VA, Williams TM, Yeh SD, True JR, Carroll SB (2006). Repeated morphological evolution through *cis*-regulatory changes in a pleiotropic gene. Nature.

[B11] Shapiro MD, Marks ME, Peichel CL, Blackman BK, Nereng KS, Jonsson B, Schluter D, Kingsley DM (2004). Genetic and developmental basis of evolutionary pelvic reduction in threespine sticklebacks. Nature.

[B12] Wittkopp PJ, Haerum BK, Clark AG (2004). Evolutionary changes in *cis *and *trans *gene regulation. Nature.

[B13] Wray GA (2003). Transcriptional regulation and the evolution of development. Int J Dev Biol.

[B14] Hammock EA, Young LJ (2005). Microsatellite instability generates diversity in brain and sociobehavioral traits. Science.

[B15] Lim MM, Wang Z, Olazabal DE, Ren X, Terwilliger EF, Young LJ (2004). Enhanced partner preference in a promiscuous species by manipulating the expression of a single gene. Nature.

[B16] Anand S, Wang WC, Powell DR, Bolanowski SA, Zhang J, Ledje C, Pawashe AB, Amemiya CT, Shashikant CS (2003). Divergence of Hoxc8 early enhancer parallels diverged axial morphologies between mammals and fishes. Proc Natl Acad Sci USA.

[B17] Khaitovich P, Weiss G, Lachmann M, Hellmann I, Enard W, Muetzel B, Wirkner U, Ansorge W, Paabo S (2004). A neutral model of transcriptome evolution. PLoS Biol.

[B18] Zhang Y, Sturgill D, Parisi M, Kumar S, Oliver B (2007). Constraint and turnover in sex-biased gene expression in the genus *Drosophila*. Nature.

[B19] Yanai I, Graur D, Ophir R (2004). Incongruent expression profiles between human and mouse orthologous genes suggest widespread neutral evolution of transcription control. OMICS.

[B20] Birney E, Stamatoyannopoulos JA, Dutta A, Guigó R, Gingeras TR, Margulies EH, Weng Z, Snyder M, Dermitzakis ET, Thurman RE, Kuehn MS, Taylor CM, Neph S, Koch CM, Asthana S, Malhotra A, Adzhubei I, Greenbaum JA, Andrews RM, Flicek P, Boyle PJ, Cao H, Carter NP, Clelland GK, Davis S, Day N, Dhami P, Dillon SC, Dorschner MO, ENCODE Project Consortium (2007). Identification and analysis of functional elements in 1% of the human genome by the ENCODE pilot project. Nature.

[B21] Yan W, Ma L, Zilinski CA, Matzuk MM (2004). Identification and characterization of evolutionarily conserved pufferfish, zebrafish, and frog orthologs of GASZ. Biol Reprod.

[B22] Chervenak AP, Basu P, Shin M, Redmond LC, Sheng G, Lloyd JA (2006). Identification, characterization, and expression pattern of the chicken EKLF gene. Dev Dyn.

[B23] Shi X, Bosenko DV, Zinkevich NS, Foley S, Hyde DR, Semina EV, Vihtelic TS (2005). Zebrafish pitx3 is necessary for normal lens and retinal development. Mech Dev.

[B24] Marza E, Barthe C, Andre M, Villeneuve L, Helou C, Babin PJ (2005). Developmental expression and nutritional regulation of a zebrafish gene homologous to mammalian microsomal triglyceride transfer protein large subunit. Dev Dyn.

[B25] Wray GA (2007). The evolutionary significance of *cis*-regulatory mutations. Nat Rev Genet.

[B26] Bejerano G, Pheasant M, Makunin I, Stephen S, Kent WJ, Mattick JS, Haussler D (2004). Ultraconserved elements in the human genome. Science.

[B27] Pennacchio LA, Ahituv N, Moses AM, Prabhakar S, Nobrega MA, Shoukry M, Minovitsky S, Dubchak I, Holt A, Lewis KD, Plajzer-Frick I, Akiyama J, De Val S, Afzal V, Black BL, Couronne O, Eisen MB, Visel A, Rubin EM (2006). *In vivo *enhancer analysis of human conserved non-coding sequences. Nature.

[B28] Siepel A, Bejerano G, Pedersen JS, Hinrichs AS, Hou M, Rosenbloom K, Clawson H, Spieth J, Hillier LW, Richards S, Weinstock GM, Wilson RK, Gibbs RA, Kent WJ, Miller W, Haussler D (2005). Evolutionarily conserved elements in vertebrate, insect, worm, and yeast genomes. Genome Res.

[B29] Woolfe A, Goodson M, Goode DK, Snell P, McEwen GK, Vavouri T, Smith SF, North P, Callaway H, Kelly K, Walter K, Abnizova I, Gilks W, Edwards YJ, Cooke JE, Elgar G (2005). Highly conserved non-coding sequences are associated with vertebrate development. PLoS Biol.

[B30] Prabhakar S, Poulin F, Shoukry M, Afzal V, Rubin EM, Couronne O, Pennacchio LA (2006). Close sequence comparisons are sufficient to identify human *cis*-regulatory elements. Genome Res.

[B31] Thomas JW, Touchman JW, Blakesley RW, Bouffard GG, Beckstrom-Sternberg SM, Margulies EH, Blanchette M, Siepel AC, Thomas PJ, McDowell JC, Maskeri B, Hansen NF, Schwartz MS, Weber RJ, Kent WJ, Karolchik D, Bruen TC, Bevan R, Cutler DJ, Schwartz S, Elnitski L, Idol JR, Prasad AB, Lee-Lin SQ, Maduro VV, Summers TJ, Portnoy ME, Dietrich NL, Akhter N, Ayele K (2003). Comparative analyses of multi-species sequences from targeted genomic regions. Nature.

[B32] Dermitzakis ET, Clark AG (2002). Evolution of transcription factor binding sites in mammalian gene regulatory regions: conservation and turnover. Mol Biol Evol.

[B33] Odom DT, Dowell RD, Jacobsen ES, Gordon W, Danford TW, MacIsaac KD, Rolfe PA, Conboy CM, Gifford DK, Fraenkel E (2007). Tissue-specific transcriptional regulation has diverged significantly between human and mouse. Nat Genet.

[B34] Oda-Ishii I, Bertrand V, Matsuo I, Lemaire P, Saiga H (2005). Making very similar embryos with divergent genomes: conservation of regulatory mechanisms of Otx between the ascidians *Halocynthia roretzi *and *Ciona intestinalis*. Development.

[B35] Sanges R, Kalmar E, Claudiani P, D'Amato M, Muller F, Stupka E (2006). Shuffling of *cis*-regulatory elements is a pervasive feature of the vertebrate lineage. Genome Biol.

[B36] Huntley S, Baggott DM, Hamilton AT, Tran-Gyamfi M, Yang S, Kim J, Gordon L, Branscomb E, Stubbs L (2006). A comprehensive catalog of human KRAB-associated zinc finger genes: insights into 27 the evolutionary history of a large family of transcriptional repressors. Genome Res.

[B37] Blekhman R, Oshlack A, Chabot AE, Smyth GK, Gilad Y (2008). Gene regulation in primates evolves under tissue-specific selection pressures. PLoS Genet.

[B38] Gilad Y, Oshlack A, Smyth GK, Speed TP, White KP (2006). Expression profiling in primates reveals a rapid evolution of human transcription factors. Nature.

[B39] Wagner GP, Lynch VJ (2008). The gene regulatory logic of transcription factor evolution. Trends Ecol Evol.

[B40] Rifkin SA, Kim J, White KP (2003). Evolution of gene expression in the *Drosophila melanogaster *subgroup. Nat Genet.

[B41] Venkatesh B, Yap WH (2005). Comparative genomics using fugu: a tool for the identification of conserved vertebrate *cis*-regulatory elements. Bioessays.

[B42] Fisher S, Grice EA, Vinton RM, Bessling SL, McCallion AS (2006). Conservation of RET regulatory function from human to zebrafish without sequence similarity. Science.

[B43] Schadt EE, Edwards SW, GuhaThakurta D, Holder D, Ying L, Svetnik V, Leonardson A, Hart KW, Russell A, Li G, Cavet G, Castle J, McDonagh P, Kan Z, Chen R, Kasarskis A, Margarint M, Caceres RM, Johnson JM, Armour CD, Garrett-Engele PW, Tsinoremas NF, Shoemaker DD (2004). A comprehensive transcript index of the human genome generated using microarrays and computational approaches. Genome Biol.

[B44] Zhang W, Morris QD, Chang R, Shai O, Bakowski MA, Mitsakakis N, Mohammad N, Robinson MD, Zirngibl R, Somogyi E, Laurin N, Eftekharpour E, Sat E, Grigull J, Pan Q, Peng WT, Krogan N, Greenblatt J, Fehlings M, Kooy D van der, Aubin J, Bruneau BG, Rossant J, Blencowe BJ, Frey BJ, Hughes TR (2004). The functional landscape of mouse gene expression. J Biol.

[B45] Chung WY, Albert R, Albert I, Nekrutenko A, Makova KD (2006). Rapid and asymmetric divergence of duplicate genes in the human gene coexpression network. BMC Bioinformatics.

[B46] Gu X, Zhang Z, Huang W (2005). Rapid evolution of expression and regulatory divergences after yeast gene duplication. Proc Natl Acad Sci USA.

[B47] Remm M, Storm CE, Sonnhammer EL (2001). Automatic clustering of orthologs and in-paralogs from pairwise species comparisons. J Mol Biol.

[B48] Hedges SB (2002). The origin and evolution of model organisms. Nat Rev Genet.

[B49] Khaitovich P, Hellmann I, Enard W, Nowick K, Leinweber M, Franz H, Weiss G, Lachmann M, Paabo S (2005). Parallel patterns of evolution in the genomes and transcriptomes of humans and chimpanzees. Science.

[B50] Khaitovich P, Enard W, Lachmann M, Paabo S (2006). Evolution of primate gene expression. Nat Rev Genet.

[B51] Hallikas O, Palin K, Sinjushina N, Rautiainen R, Partanen J, Ukkonen E, Taipale J (2006). Genome-wide prediction of mammalian enhancers based on analysis of transcription-factor binding affinity. Cell.

[B52] Sandelin A, Alkema W, Engstrom P, Wasserman WW, Lenhard B (2004). JASPAR: an open-access database for eukaryotic transcription factor binding profiles. Nucleic Acids Res.

[B53] Whitehead A, Crawford DL (2006). Variation within and among species in gene expression: raw material for evolution. Mol Ecol.

[B54] Stuart JM, Segal E, Koller D, Kim SK (2003). A gene-coexpression network for global discovery of conserved genetic modules. Science.

[B55] Meiklejohn CD, Parsch J, Ranz JM, Hartl DL (2003). Rapid evolution of male-biased gene expression in *Drosophila*. Proc Natl Acad Sci USA.

[B56] Zapata A, Diez B, Cejalvo T, Gutierrez-de Frias C, Cortes A (2006). Ontogeny of the immune system of fish. Fish Shellfish Immunol.

[B57] Wilson MD, Barbosa-Morais NL, Schmidt D, Conboy CM, Vanes L, Tybulewicz VL, Fisher EM, Tavare S, Odom DT (2008). Species-specific transcription in mice carrying human chromosome 21. Science.

[B58] Bustamante CD, Fledel-Alon A, Williamson S, Nielsen R, Hubisz MT, Glanowski S, Tanenbaum DM, White TJ, Sninsky JJ, Hernandez RD, Civello D, Adams MD, Cargill M, Clark AG (2005). Natural selection on protein-coding genes in the human genome. Nature.

[B59] Lopez-Bigas N, De S, Teichmann SA (2008). Functional protein divergence in the evolution of *Homo sapiens*. Genome Biol.

[B60] Berger MF, Badis G, Gehrke AR, Talukder S, Philippakis AA, Pena-Castillo L, Alleyne TM, Mnaimneh S, Botvinnik OB, Chan ET, Khalid F, Zhang W, Newburger D, Jaeger SA, Morris QD, Bulyk ML, Hughes TR (2008). Variation in homeodomain DNA binding revealed by high-resolution analysis of sequence preferences. Cell.

[B61] Luscombe NM, Thornton JM (2002). Protein-DNA interactions: amino acid conservation and the effects of mutations on binding specificity. J Mol Biol.

[B62] Segal E, Raveh-Sadka T, Schroeder M, Unnerstall U, Gaul U (2008). Predicting expression patterns from regulatory sequence in *Drosophila *segmentation. Nature.

[B63] Conservation of Core Gene Expression in Vertebrate Tissues: Supplementary Data. http://hugheslab.ccbr.utoronto.ca/supplementary-data/vertebrate_expression.

[B64] Hubbard TJ, Aken BL, Beal K, Ballester B, Caccamo M, Chen Y, Clarke L, Coates G, Cunningham F, Cutts T, Down T, Dyer SC, Fitzgerald S, Fernandez-Banet J, Graf S, Haider S, Hammond M, Herrero J, Holland R, Howe K, Howe K, Johnson N, Kahari A, Keefe D, Kokocinski F, Kulesha E, Lawson D, Longden I, Melsopp C, Megy K (2007). Ensembl 2007. Nucleic Acids Res.

[B65] Su AI, Wiltshire T, Batalov S, Lapp H, Ching KA, Block D, Zhang J, Soden R, Hayakawa M, Kreiman G, Cooke MP, Walker JR, Hogenesch JB (2004). A gene atlas of the mouse and human protein-encoding transcriptomes. Proc Natl Acad Sci USA.

[B66] Kent WJ (2002). BLAT – the BLAST-like alignment tool. Genome Res.

[B67] O'Brien KP, Remm M, Sonnhammer EL (2005). Inparanoid: a comprehensive database of eukaryotic orthologs. Nucleic Acids Res.

[B68] Altschul SF, Gish W, Miller W, Myers EW, Lipman DJ (1990). Basic local alignment search tool. J Mol Biol.

[B69] Alexeyenko A, Tamas I, Liu G, Sonnhammer EL (2006). Automatic clustering of orthologs and inparalogs shared by multiple proteomes. Bioinformatics.

[B70] Kasprzyk A, Keefe D, Smedley D, London D, Spooner W, Melsopp C, Hammond M, Rocca-Serra P, Cox T, Birney E (2004). EnsMart: a generic system for fast and flexible access to biological data. Genome Res.

[B71] Huber W, von Heydebreck A, Sultmann H, Poustka A, Vingron M (2002). Variance stabilization applied to microarray data calibration and to the quantification of differential expression. Bioinformatics.

[B72] Schug J, Schuller WP, Kappen C, Salbaum JM, Bucan M, Stoeckert CJ (2005). Promoter features related to tissue specificity as measured by Shannon entropy. Genome Biol.

[B73] UCSC Genome Bioinformatics. http://genome.ucsc.edu.

[B74] Electronic Supplement: Ultraconserved Elements in the Human Genome. http://www.soe.ucsc.edu/~jill/ultra.html.

[B75] Finn RD, Tate J, Mistry J, Coggill PC, Sammut SJ, Hotz HR, Ceric G, Forslund K, Eddy SR, Sonnhammer EL, Bateman A (2008). The Pfam protein families database. Nucleic Acids Res.

[B76] Brudno M, Do CB, Cooper GM, Kim MF, Davydov E, Green ED, Sidow A, Batzoglou S (2003). LAGAN and Multi-LAGAN: efficient tools for large-scale multiple alignment of genomic DNA. Genome Res.

[B77] Brudno M, Malde S, Poliakov A, Do CB, Couronne O, Dubchak I, Batzoglou S (2003). Glocal alignment: finding rearrangements during alignment. Bioinformatics.

[B78] Schwartz S, Kent WJ, Smit A, Zhang Z, Baertsch R, Hardison RC, Haussler D, Miller W (2003). Human-mouse alignments with BLASTZ. Genome Res.

