# Supplementary material for: Conservation of core gene expression in vertebrate tissues
Source: J Biol. 2009 Apr 16;8(3):33. doi: 10.1186/jbiol130 (PMC2689434; doi:10.1186/jbiol130)
Supplement: Additional data file 1 — The tissues at the top, highlighted in color, are those considered to be among the ten common tissue types. Those with identical coloring were combined (by averaging normalized intensities) for the analysis of conservation of gene expression among the ten common tissues. [file jbiol130-S1.pdf]

Tissues assayed			
Chicken (*Gallus gallus*)	Frog (*Xenopus tropicalis*)	Pufferfish (*Tetraoodon nigroviridis*)	
Cerebral cortex	Brain	Brain	Common tissues (published data also in Human and Mouse)
Cerebellum			Common tissues (published data also in Human and Mouse)
Eye	Eye	Eye	Common tissues (published data also in Human and Mouse)
Heart	Heart	Heart	Common tissues (published data also in Human and Mouse)
Intestine	Large intestine	Intestine	Common tissues (published data also in Human and Mouse)
	Small intestine		Common tissues (published data also in Human and Mouse)
Kidney	Kidney	Kidney	Common tissues (published data also in Human and Mouse)
Liver	Liver	Liver	Common tissues (published data also in Human and Mouse)
Skeletal muscle	Skeletal muscle	Skeletal muscle (red)	Common tissues (published data also in Human and Mouse)
		Skeletal muscle (white)	Common tissues (published data also in Human and Mouse)
Spleen	Spleen	Spleen	Common tissues (published data also in Human and Mouse)
Stomach	Stomach	Stomach	Common tissues (published data also in Human and Mouse)
Testis	Testis	Testis	Common tissues (published data also in Human and Mouse)
Bursa of Fabricius	Cartilage	Beak (fused teeth)	Additional tissues (may or may not have data in all other organisms)
Bone marrow + Femur	Esophagus	Calvaria	Additional tissues (may or may not have data in all other organisms)
Gallbladder	Fat body	Connective tissue	Additional tissues (may or may not have data in all other organisms)
Gizzard	Gallbladder	Fin	Additional tissues (may or may not have data in all other organisms)
Lung	Femur	Gallbladder	Additional tissues (may or may not have data in all other organisms)
Ovary	Lung	Gill	Additional tissues (may or may not have data in all other organisms)
Oviduct	Ovary	Ovary	Additional tissues (may or may not have data in all other organisms)
Skin	Oviduct	Skin	Additional tissues (may or may not have data in all other organisms)
Thymus	Skin	Swimbladder	Additional tissues (may or may not have data in all other organisms)

Additional file 1
