# Supplementary figures and images for: Conservation of core gene expression in vertebrate tissues
Source: J Biol. 2009 Apr 16;8(3):33. doi: 10.1186/jbiol130 (PMC2689434; doi:10.1186/jbiol130)

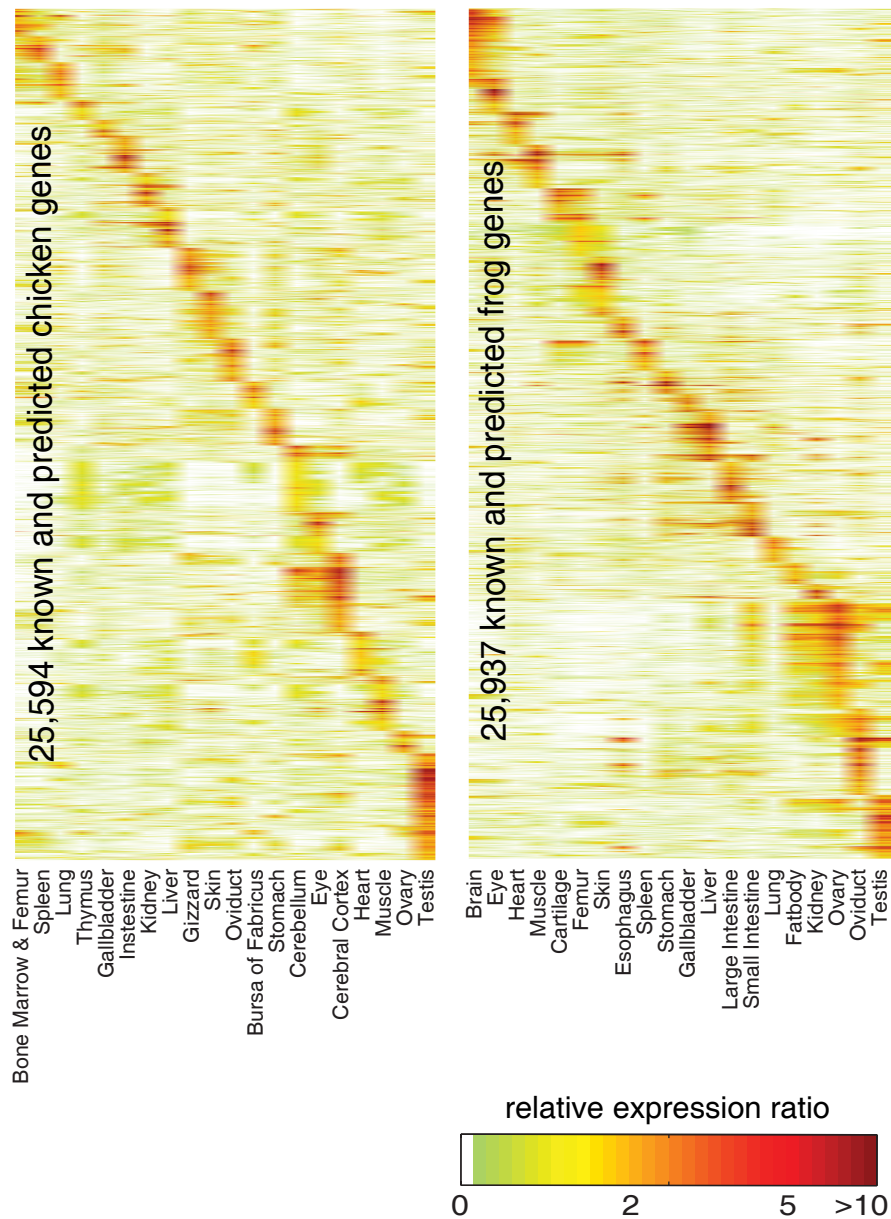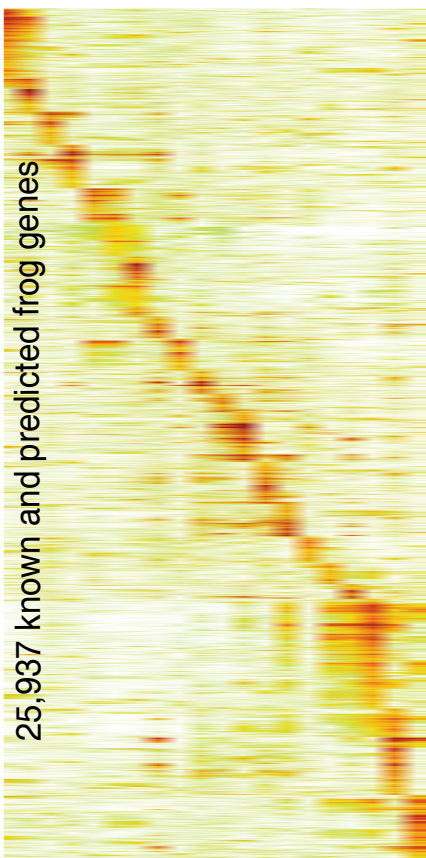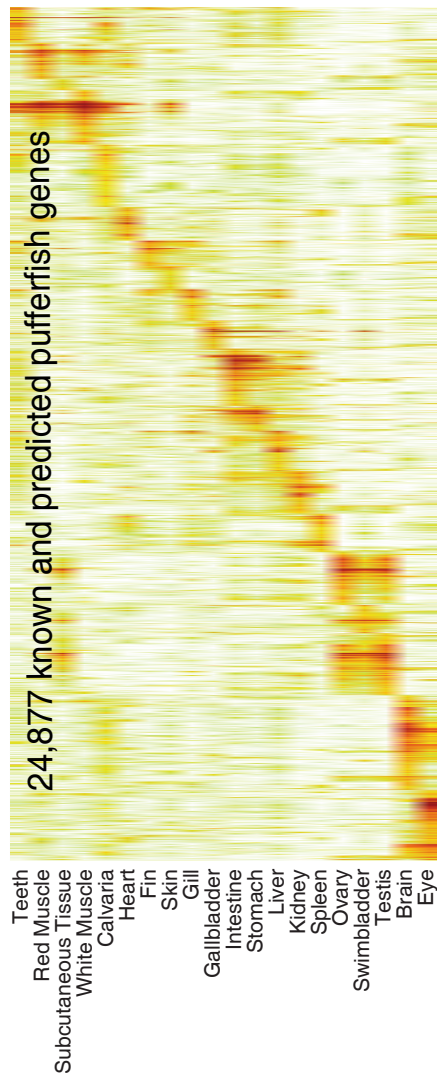

Additional file 2

Supplement: Additional data file 2 — Clustergrams show the microarray datasets in chicken, frog and pufferfish, displayed as relative expression ratio (see Materials and methods) of each gene within each of the 20 tissues profiled. Rows and columns were ordered independently for each dataset, and high-level branches broken and rearranged to obtain a diagonal appearance as described in [44]. [file jbiol130-S2.pdf]

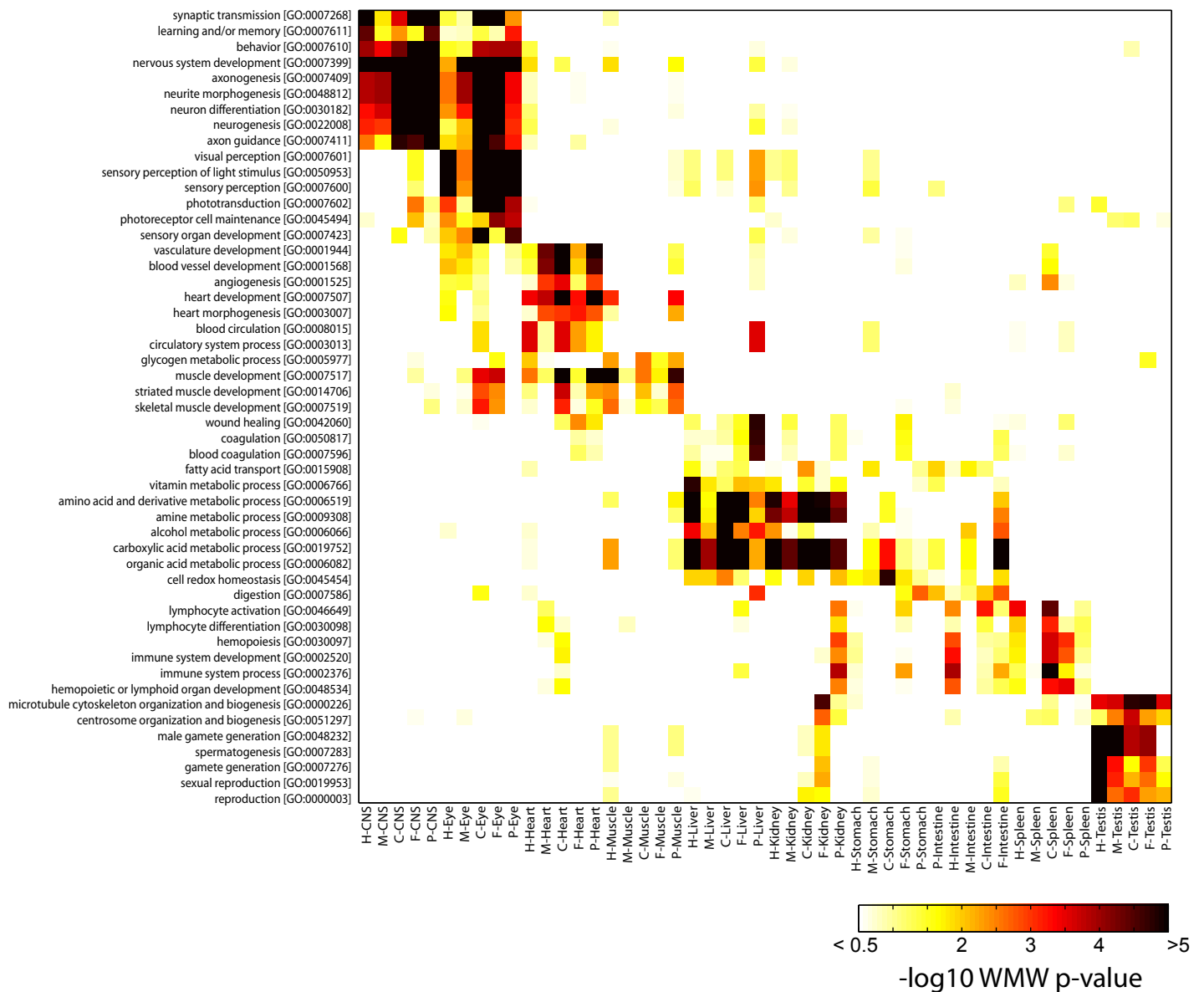

Additional file 4

Supplement: Additional data file 4 — Selected GO biological process categories enriched amongst genes highly expressed within each of the ten common tissues in each species are shown. The tissue and GO category order were manually arranged in the heat map. (A full matrix of WMW scores is given in Additional data file 13.) [file jbiol130-S4.pdf]

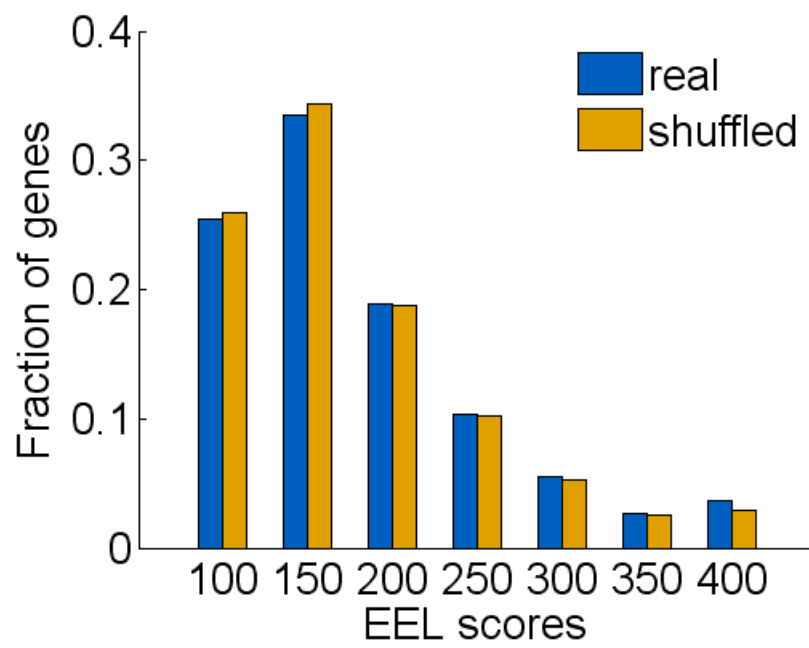

Additional File 9

Supplement: Additional data file 9 — Cumulative distribution of EEL scores for real and permuted orthology between human and pufferfish. [file jbiol130-S9.pdf]

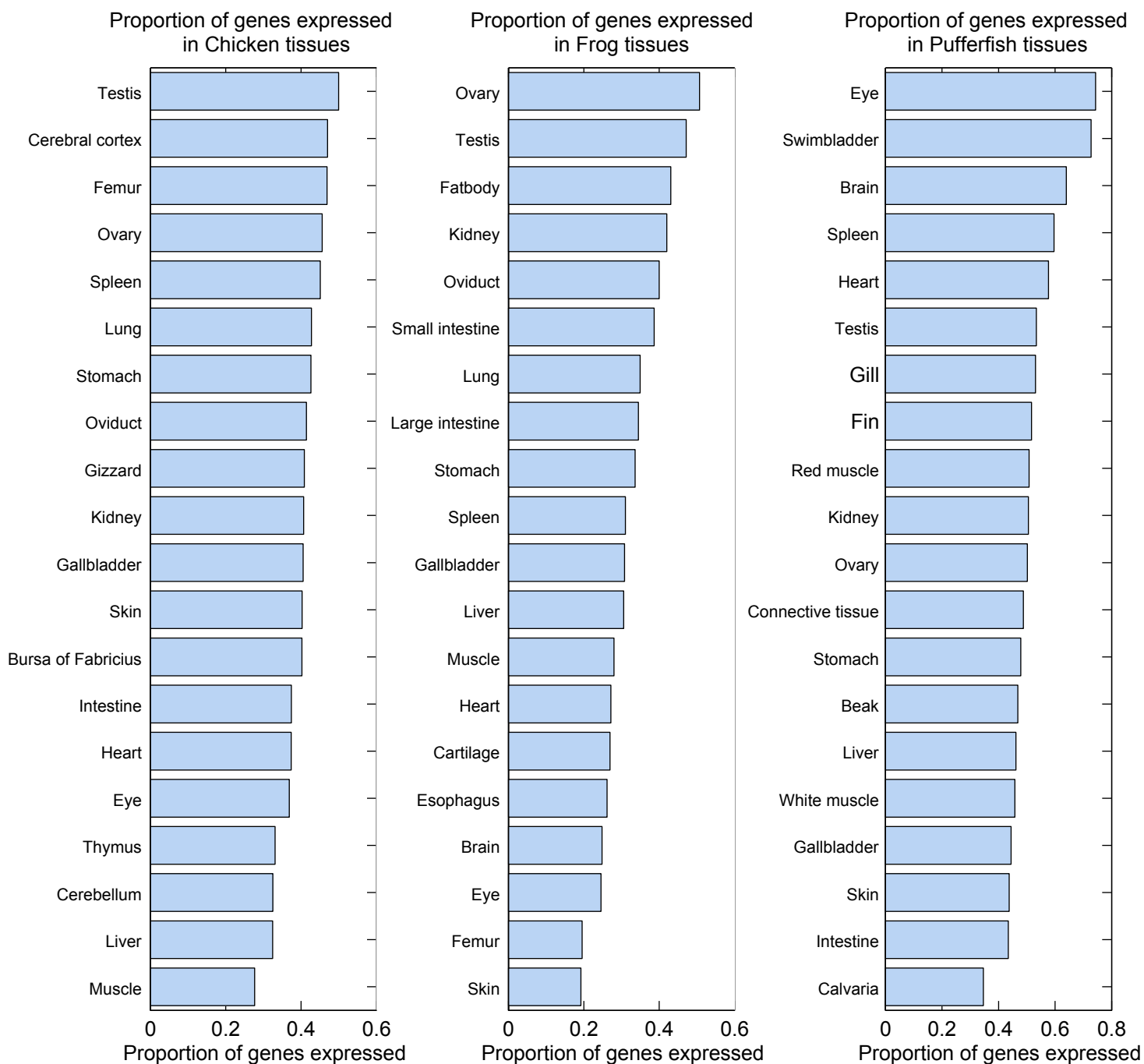

Supplement: Additional data file 10 — Breakdown of the proportion of all genes in each species that are expressed within each tissue. [file jbiol130-S10.pdf]

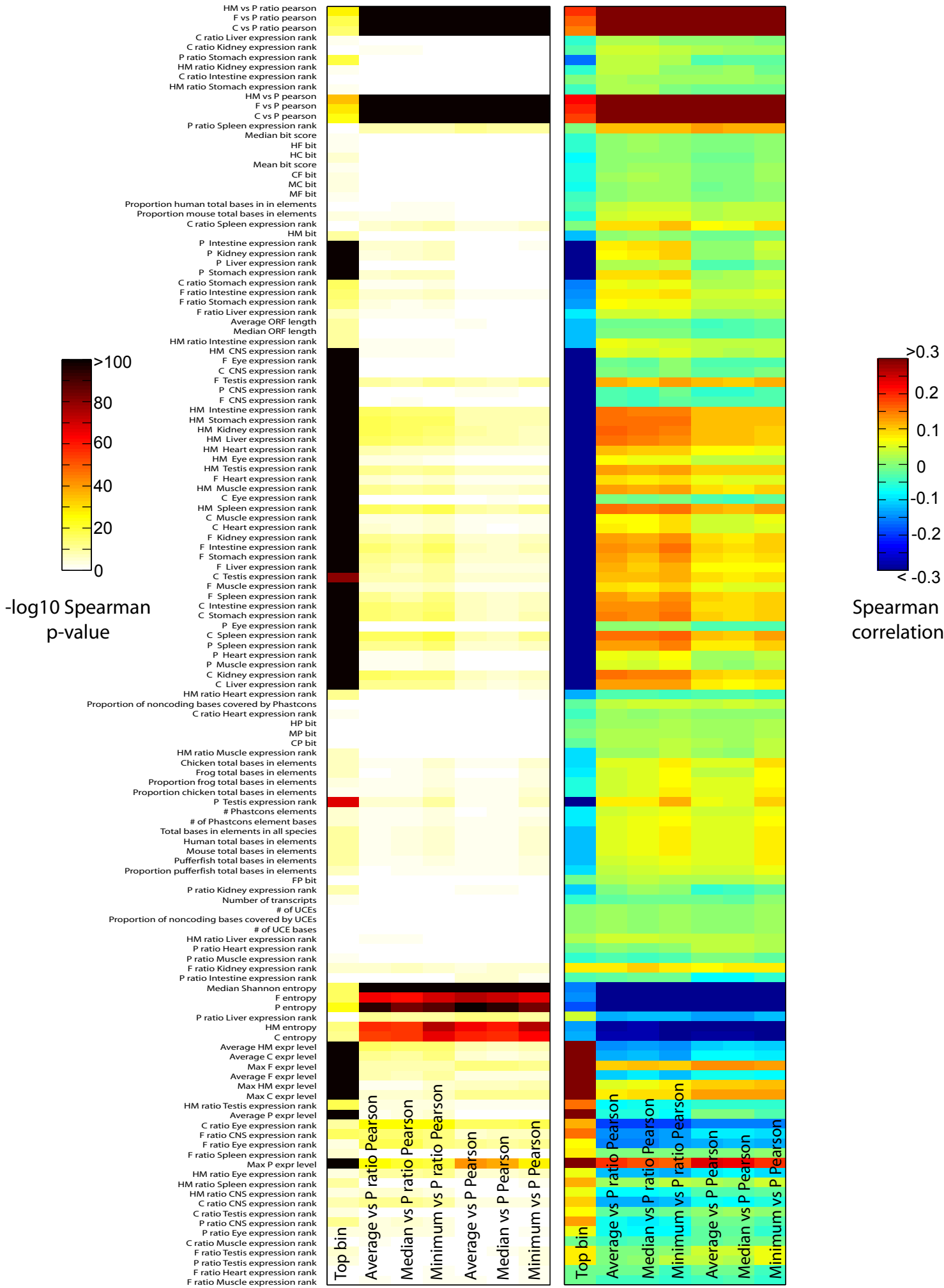

Supplement: Additional data file 12 — Clustergrams showing Spearman correlations and p-values for comparisons of gene expression conservation versus other gene properties. [file jbiol130-S12.pdf]
