# Supplementary material for: Conservation of core gene expression in vertebrate tissues
Source: J Biol. 2009 Apr 16;8(3):33. doi: 10.1186/jbiol130 (PMC2689434; doi:10.1186/jbiol130)
Supplement: Additional data file 3 — Dendrogram of correlations among ten common tissues, using 1 – Pearson correlation and average linkage over 3,074 genes. [file jbiol130-S3.pdf]

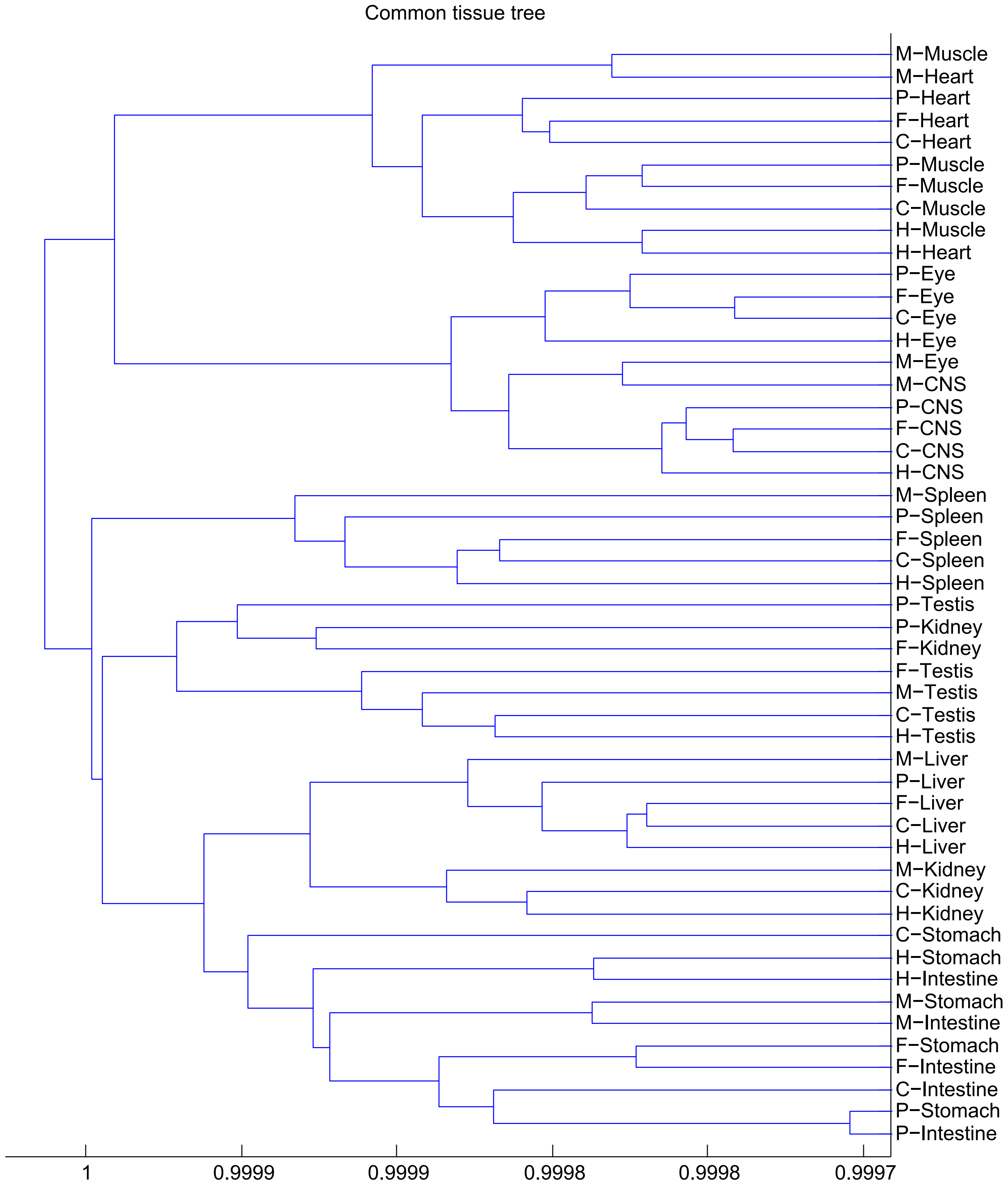

Dendrogram derived from 1- Pearson correlation of expression ratio vectors across all 3,074 measured genes using average linkage.

Additional file 3
