# Supplementary material for: Conservation of core gene expression in vertebrate tissues
Source: J Biol. 2009 Apr 16;8(3):33. doi: 10.1186/jbiol130 (PMC2689434; doi:10.1186/jbiol130)
Supplement: Additional data file 6 — The cumulative distributions show the proportion of all 3,074 genes with Pearson r (normalized intensities) below the value shown on the horizontal axis, for real orthologs (green) and randomly matched genes (blue). [file jbiol130-S6.pdf]

Pairwise species comparisons of expression similarity between orthologs (3,074) and randomly-matched genes (10,000) on expression ratios

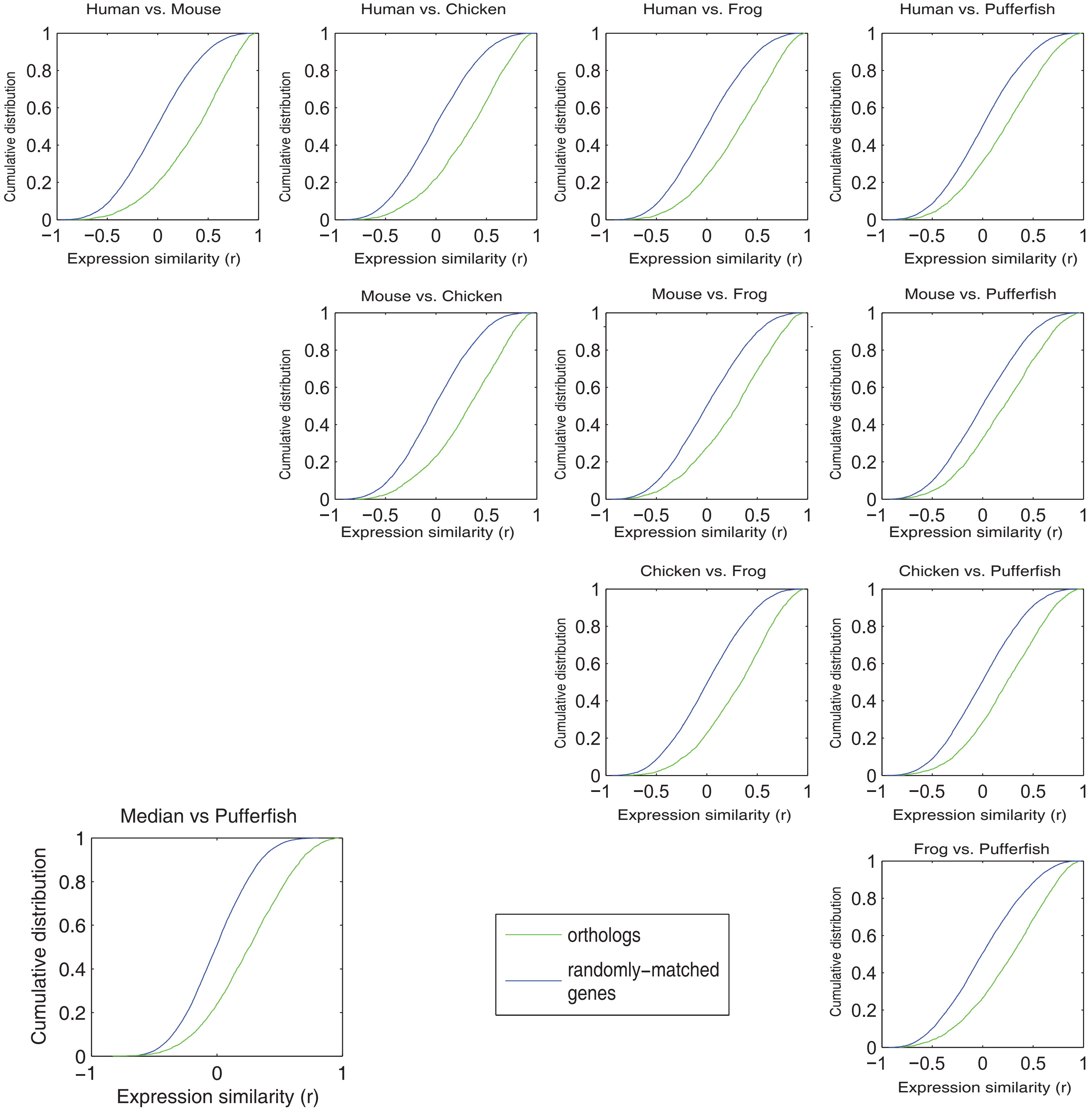

Additional file 6
